# Dental Pathology

**Published:** 1883-04

**Authors:** 


### DENTAL PATHOLOGY.

Dr. W. D. Miller, of Berlin, is about to commence a new series of observations and experiments in dental pathology in Koch's laboratory, and will have the benefit of the experience advice and co-operation, of that famous observer. The dental profession in America will reap the earliest benefits from Dr. Miller's studies, and we hope to be the medium of their communication. It is little more than a year since he was first introduced to American readers, and during that time he has secured an abiding place in our literature. Next month we shall present a valuable paper from his pen upon Dental Caries.

